# How mentor teachers' transformational leadership during practicum influences pre-service teachers' scholarship of teaching and learning: the mediating role of teacher self-efficacy and the moderating role of work engagement

**DOI:** 10.3389/fpsyg.2026.1861802

**Published:** 2026-06-22

**Authors:** Ping Yong, Xiaotian Fang

**Affiliations:** College of International Education, Sichuan International Studies University, Chongqing, China

**Keywords:** mentor teachers, pre-service teachers, scholarship of teaching and learning, teacher self-efficacy, transformational leadership, work engagement

## Abstract

**Introduction:**

In the context of teacher education reform and the pursuit of high-quality teacher preparation, it is important to examine the mechanisms underlying the development of pre-service teachers' scholarship of teaching and learning (SoTL). However, the mechanisms through which practicum mentors' leadership behaviors shape pre-service teachers' SoTL remain insufficiently understood.

**Methods:**

Drawing on transformational leadership theory and social cognitive theory, this study developed a moderated mediation model to investigate how mentor teachers' transformational leadership influences pre-service teachers' SoTL, with teacher self-efficacy (TSE) as a mediator and work engagement as a moderator. Data were collected from 613 pre-service teachers enrolled in Master of Education programs at five universities in western China. Structural equation modeling and PROCESS Model 14 were employed for data analysis.

**Results:**

Mentor teachers' transformational leadership significantly and positively predicted both pre-service teachers' SoTL and TSE. TSE partially mediated the relationship between transformational leadership and SoTL. In addition, work engagement significantly moderated the relationship between TSE and SoTL, such that the positive effect of TSE on SoTL was stronger at higher levels of work engagement.

**Discussion:**

These findings suggest that mentor teachers serve not only as practical supervisors during practicum but also as important contextual sources of pre-service teachers' TSE formation and professional competence development. This study extends transformational leadership theory to informal mentoring contexts in teacher education and provides practical implications for optimizing practicum supervision systems, strengthening mentor training, and promoting pre-service teachers' professional development.

## 1 Introduction

Against the backdrop of global educational reform and the increasing professionalization of teaching, the development of pre-service teacher competencies has attracted growing scholarly attention. For graduate students in teacher education programs, the practicum represents a pivotal transitional period—one in which they shift from the role of student to that of teacher, and in which they begin to form their pedagogical beliefs, accumulate practical teaching experience, and develop core professional competencies. Central to this developmental process is the capacity for Scholarship of Teaching and Learning (SoTL), which refers to the systematic use of inquiry-based thinking and research methods to reflect on, continuously improve, and innovate one's teaching practice (Boyer, 1990; Yong and Peng, 2025). Yet the practicum period poses particular challenges for pre-service teachers: situated between theoretical preparation and full professional immersion, they are still constructing the cognitive and motivational resources that SoTL requires. Understanding what contextual supports and psychological conditions facilitate SoTL formation during this stage is therefore both theoretically important and practically urgent.

Among the contextual factors shaping pre-service teacher development, practicum mentors occupy a particularly influential role. Research has consistently demonstrated that mentoring relationships promote graduate students' innovative behavior, research competence, and career development (Han et al., 2022; Ren and Li, 2024). Within teacher education specifically, studies have highlighted the critical functions of mentor interaction, observation-based feedback, and practical support in fostering professional learning during the practicum (Goldshaft and Sjølie, 2024; Orland-Barak and Wang, 2021). In the Chinese teacher education context, these mentors—typically referred to as school-based practicum mentors or cooperating teachers—are experienced in-service teachers from partner schools who provide classroom guidance, practical supervision, and professional feedback to pre-service teachers throughout the practicum period. Although they hold no formal administrative authority, their mentoring behaviors—including offering an inspiring vision, providing individualized care, encouraging experimentation, and giving reflective feedback—align closely with the defining features of transformational leadership (Bass, 1985, 1995). Accordingly, conceptualizing school-based practicum mentors as informal transformational leaders offers a theoretically grounded lens for examining how their guidance influences pre-service teachers' SoTL development.

Examining the direct effect of transformational leadership on SoTL, however, does not fully account for the psychological processes through which such influence operates. Social cognitive theory posits that self-efficacy beliefs—individuals' judgments about their capacity to organize and execute the actions required to produce given outcomes—are a central regulatory mechanism governing behavioral choice, effort, and persistence (Bandura, 1997). In teacher education contexts, teacher self-efficacy (TSE) is specifically defined as an individual's belief in their ability to organize and effectively carry out the tasks required to teach successfully (Tschannen-Moran and Hoy, 2001). Because pre-service teachers' professional identities and practical experiences are still consolidating during the practicum, their TSE is particularly susceptible to the influence of mentor support, instructional feedback, and the broader practice environment. TSE is therefore likely to function as a key psychological mechanism linking mentors' transformational leadership to pre-service teachers' SoTL development.

That said, efficacy beliefs do not automatically translate into sustained professional practice. Work engagement—defined as a positive, fulfilling, work-related state characterized by vigor, dedication, and absorption (Schaufeli et al., 2006)—reflects the extent to which individuals actively and persistently invest their psychological resources in task-related activities. Even when pre-service teachers hold strong TSE beliefs, a low level of engagement during the practicum may prevent them from sustaining the reflective inquiry, pedagogical innovation, and research-oriented practice that SoTL demands. Conversely, when engagement is high, positive efficacy beliefs are more likely to be channeled into specific, enduring SoTL behaviors. Work engagement may therefore constitute an important boundary condition moderating the extent to which TSE translates into SoTL.

To address these gaps, the present study integrates transformational leadership theory with social cognitive theory to construct and test a moderated mediation model examining how school-based practicum mentors' transformational leadership influences pre-service teachers' SoTL. Specifically, we aim to investigate: (1) whether transformational leadership significantly promotes pre-service teachers' SoTL; (2) whether TSE mediates this relationship; and (3) whether work engagement moderates the TSE → SoTL pathway, such that higher engagement strengthens the effect of TSE on SoTL. By addressing these questions, the present study makes three contributions. Theoretically, it extends the application of transformational leadership theory to informal mentoring contexts in pre-service teacher education, and advances understanding of the psychological mechanisms underpinning SoTL development. Empirically, it introduces work engagement as a boundary condition in the TSE-to-SoTL conversion process, offering a more dynamic account of how efficacy beliefs are translated into sustained professional practice. Practically, the findings provide evidence-based guidance for the design of practicum supervision systems and mentor development programs aimed at cultivating SoTL in pre-service teachers.

## 2 Literature review and hypotheses developments

### 2.1 Transformational leadership of school-based practicum mentors and pre-service teachers' SoTL

SoTL emphasizes the systematic use of inquiry-based thinking to examine teaching practice, and the continuous improvement of teaching quality through sustained reflection, evidence-informed decision making, and pedagogical innovation (Boyer, 1990). For pre-service teachers in the practicum stage, SoTL not only reflects the depth of their engagement with and reflection on teaching, but is also substantially shaped by the quality of practical experience and external mentoring support they receive.

Among the contextual factors influencing pre-service teacher development, school-based practicum mentors serve as a critical source of professional support. Research has consistently shown that mentoring relationships significantly affect student teachers‘ learning experiences, classroom adaptation, and capacity for professional reflection, with the quality of feedback, modes of interaction, and levels of support having direct consequences for practicum outcomes and the development of professional judgment. Compared with general supportive behaviors, the behaviors that practicum mentors characteristically enact—including offering an inspiring vision, encouraging experimentation, stimulating intellectual inquiry, and providing individualized care—are more appropriately understood through the lens of transformational leadership. (Bass 1985, 1995) proposed that transformational leadership activates individual potential and enhances initiative and creativity through four key dimensions: idealized influence, inspirational motivation, intellectual stimulation, and individualized consideration (Bass and Riggio, 2006). Empirical research in higher education contexts has further demonstrated that mentor behaviors reflecting idealized influence, motivational support, and intellectual stimulation significantly enhance students' proactive engagement and innovative tendencies, and produce incremental positive effects on their career commitment and satisfaction (Fisher, 2023; Gu et al., 2015; Scandura and Williams, 2004). More broadly, transformational leadership has been shown to positively predict teacher innovativeness and professional performance across educational settings (Hidayat and Patras, 2024; Leithwood and Jantzi, 2006).

This perspective is equally well-supported in the teacher education literature. (Kuswandono 2017) found that school-based practicum mentors frequently serve as role models through professional demonstration and pedagogical modeling, with high-quality mentoring playing a pivotal role in student teachers' professional growth. (Galamay-Cachola et al. 2018) further demonstrated that practicum mentors typically exhibit behaviors characterized by motivational encouragement, guided inquiry, and emotional support—collectively creating a mentoring climate that closely mirrors the defining features of transformational leadership—and that such a climate effectively promotes student teachers' reflective practice and professional development (Kuhn et al., 2024). Taken together, these findings suggest that when school-based practicum mentors exhibit strong transformational leadership, they are more likely to directly foster pre-service teachers' SoTL by stimulating active learning, encouraging pedagogical reflection, and supporting strategic innovation in teaching. It should be noted that most existing studies on transformational leadership and SoTL have been conducted in Western contexts; the present study extends this line of inquiry to the Chinese practicum setting, where mentor–mentee relationships are shaped by distinct institutional and cultural norms (Orland-Barak and Wang, 2021).

The focus on transformational leadership in this study was theoretically motivated by the nature of practicum mentoring. Compared with general mentor support, transformational leadership provides a more specific framework for explaining how mentors inspire professional vision, stimulate pedagogical inquiry, offer individualized consideration, and encourage experimentation in teaching—dimensions that closely correspond to the developmental tasks faced by pre-service teachers during practicum. The present study does not assume that transformational leadership exhausts all possible forms of mentor influence; rather, it uses this framework to examine a specific set of leadership-like mentoring behaviors that are particularly relevant to SoTL development. While transactional and instructional leadership frameworks also offer relevant perspectives on mentoring, transformational leadership was prioritized because its emphasis on inspiration, intellectual stimulation, and individualized consideration more directly addresses the motivational and identity-forming dimensions of pre-service teacher development during practicum (Bass and Riggio, 2006).

Based on this reasoning, the following hypothesis is proposed:

H1: School-based practicum mentors' transformational leadership is positively associated with pre-service teachers' SoTL.

### 2.2 The mediating role of teacher self-efficacy

While school-based practicum mentors' transformational leadership may directly promote pre-service teachers' SoTL, it is unlikely to operate solely through external influence; the underlying psychological mechanisms warrant further examination. Social cognitive theory posits that self-efficacy—an individual's belief in their capacity to organize and execute the actions required to accomplish specific tasks—is a critical psychological mechanism linking environmental factors to behavioral outcomes, by shaping behavioral choices, determining the level of effort invested, and sustaining persistence in the face of difficulty (Bandura, 1997). In the context of teacher education, teacher self-efficacy (TSE) is specifically defined as an individual's belief in their ability to organize classroom instruction, manage learning environments, and promote student learning (Tschannen-Moran and Hoy, 2001). Crucially, self-efficacy is not a fixed trait; rather, it develops progressively through multiple information sources, including mastery experiences, vicarious experiences, social persuasion, and physiological states (Bandura, 1997).

There is substantial empirical support for the pathway from transformational leadership to TSE. For pre-service teachers in the practicum stage, school-based mentors are positioned to influence their judgments about their own teaching capabilities through instructional modeling, evaluative feedback, encouragement of risk-taking, and emotional support. When mentors exhibit strong transformational leadership characteristics, pre-service teachers are more likely to receive affirming feedback and experience validation of their emerging competencies, thereby enhancing their TSE. (Lefteri and Menon 2025) demonstrated in a school-based study that leadership behaviors characterized by visionary guidance and individualized consideration significantly predicted improvements in teachers' self-efficacy. Converging evidence further indicates that mentor support and leadership behaviors more broadly can substantially strengthen teachers' and graduate students' efficacy beliefs, with downstream effects on innovative behavior and professional development (Gu et al., 2015; Ren and Li, 2024; Yilmaz and Tore, 2025). Accordingly, school-based practicum mentors' transformational leadership is likely to constitute an important external source for the formation of pre-service teachers' TSE.

At the same time, TSE may in turn facilitate the development of SoTL. Pre-service teachers with higher levels of TSE are more likely to approach pedagogical challenges proactively, sustain their investment in lesson preparation, experiment with novel instructional strategies, and maintain the motivation to reflect on and improve their teaching practice (Zee and Koomen, 2016). (Cai and Tang 2021) demonstrated that TSE mediates the relationship between school support and teacher innovation, suggesting that self-efficacy functions as a psychological conduit through which external resources are translated into concrete professional behaviors, particularly during practicum placements (Ma et al., 2022). For pre-service teachers at the outset of their professional careers, TSE is therefore likely to serve as an important bridge between external mentoring support and sustained professional practice—and, by extension, as a key psychological channel through which mentors' transformational leadership shapes SoTL development. It is worth noting, however, that the TSE–performance link is not uniformly strong across contexts; Klassen and Tze's (2014) meta-analysis found a significant but modest overall effect size, suggesting that boundary conditions—such as work engagement—may play an important role in determining when TSE translates into professional behavior.

Based on the foregoing analysis, the following hypotheses are proposed:

H2: School-based practicum mentors' transformational leadership is positively associated with pre-service teachers' TSE.

H3: TSE mediates the relationship between school-based practicum mentors' transformational leadership and pre-service teachers' SoTL.

### 2.3 The moderating role of work engagement

Although TSE provides an important psychological foundation for pre-service teachers to engage in pedagogical reflection, innovative experimentation, and inquiry-oriented practice, positive efficacy beliefs do not automatically translate into sustained professional behavior. For efficacy beliefs to manifest as genuine SoTL development, individuals must invest sufficient psychological energy and behavioral effort in their practical tasks. Work engagement is a key variable for understanding this translation process. (Schaufeli et al. 2006) define work engagement as a positive, fulfilling, work-related state characterized by vigor, dedication, and absorption, reflecting the extent to which individuals actively and persistently invest themselves in task-related activities.

In the teacher education context, work engagement can be understood as pre-service teachers' active psychological investment in practicum teaching, lesson preparation, reflective practice, and other professional tasks. A growing body of research indicates that work engagement is significantly associated with innovative behavior, work performance, and professional development, and may play a pivotal role in facilitating the conversion of efficacy beliefs into actual behavioral outcomes (Cai et al., 2022; Johnson, 2022; Uppathampracha and Liu, 2022; Hassan et al., 2024).

For pre-service teachers, TSE strengthens the judgment that “I am capable of doing this well”; however, this judgment is more likely to translate into sustained pedagogical reflection, practical improvement, and innovative exploration only when individuals maintain a high level of engagement with their teaching tasks. In other words, under conditions of high work engagement, pre-service teachers are more likely to channel positive efficacy beliefs into specific and enduring SoTL behaviors; conversely, when engagement is low, the facilitative effect of TSE on SoTL may be attenuated. Work engagement is therefore hypothesized to moderate—and specifically to amplify—the positive influence of TSE on SoTL, constituting an important boundary condition in this translation process.

Accordingly, the following hypothesis is proposed:

H4: Work engagement positively moderates the relationship between TSE and SoTL, such that the positive effect of TSE on SoTL is stronger when work engagement is higher.

### 2.4 Theoretical integration and research model

**Figure 1:**
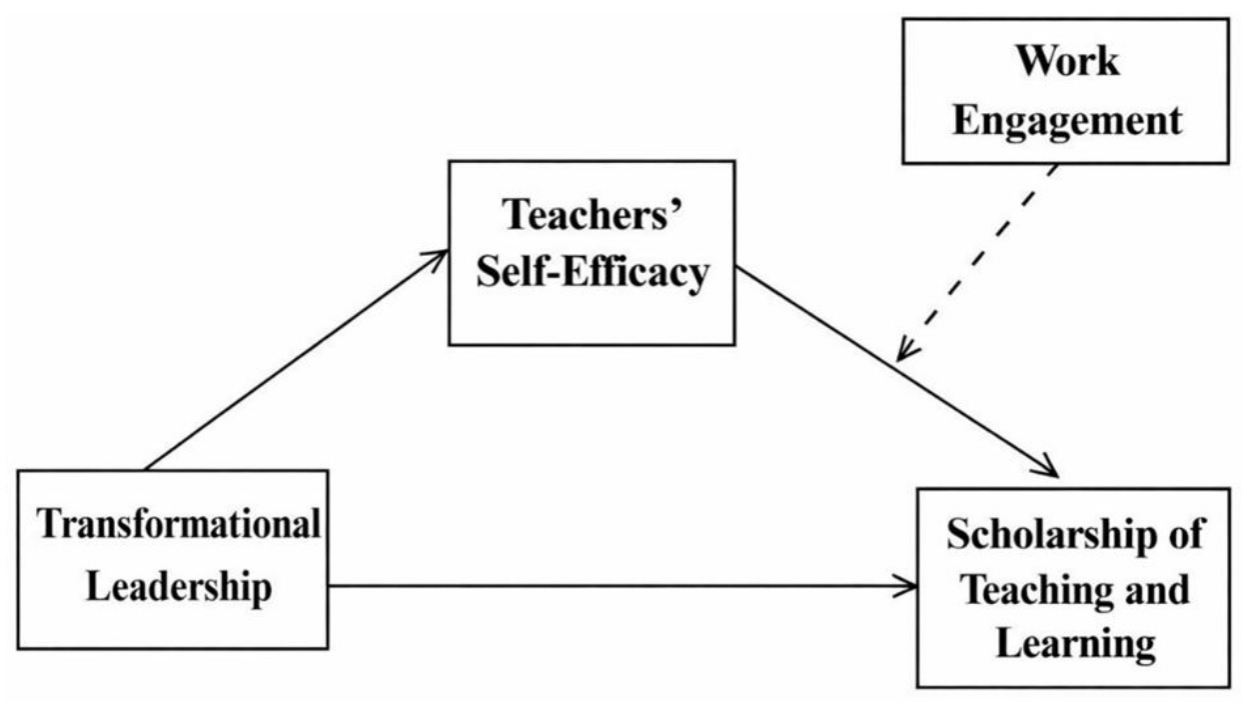
Conceptual model of the study.

Drawing on transformational leadership theory and social cognitive theory, the present study constructs a moderated mediation model to elucidate the mechanisms through which school-based practicum mentors' transformational leadership influences pre-service teachers' SoTL. Specifically, mentors‘ transformational leadership is proposed to exert both a direct effect on SoTL and an indirect effect mediated by TSE: mentor behaviors that inspire vision, stimulate intellectual inquiry, and provide individualized support are expected to strengthen pre-service teachers' efficacy beliefs, which in turn promote sustained engagement in reflective, innovative, and inquiry-oriented teaching practice. Additionally, work engagement is proposed to moderate the TSE → SoTL pathway, amplifying the extent to which efficacy beliefs are translated into SoTL behaviors under conditions of higher engagement. The full research model is depicted in Figure 1.

## 3 Methods

This study employed a quantitative research design, with data collected via a self-report questionnaire survey. The research procedure comprised two phases: a pilot phase involving scale adaptation and small-sample testing, and a main survey phase involving full-scale data collection and statistical analysis. To enhance the contextual fit and psychometric quality of the measurement instruments, the research team conducted a pilot survey prior to formal data collection, revised scale items based on pilot feedback, and subsequently proceeded to the main data collection and analysis.

### 3.1 Participants and Procedures

Participants were graduate students enrolled in Master of Education programs at five universities in western China, all of whom had completed supervised practicum placements under the guidance of school-based practicum mentors. Because practicum supervision in these programs is primarily arranged during the second and third years of study, the sample was restricted to students at these two stages of their graduate training.

Purposive cluster sampling was employed, with intact classes serving as the sampling units. A total of 613 valid questionnaires were obtained. The sample spanned multiple teacher education specializations, including Early Childhood Education, Primary Education, and Secondary Education, and exhibited diversity in terms of gender, year of study, and place of origin, supporting the representativeness of the findings. Demographic characteristics of the sample are presented in Table 1.

**Table 1:** Descriptive statistics of the sample.

Demographic characteristics	Category	Frequency (*n*)	Percentage (%)	Cumulative (%)
Gender	Male	115	19.5	19.5
	Female	498	80.5	100
Grade level	Second-year graduate student	324	52.8	52.8
	Third-year graduate student	289	47.2	100
Place of origin	Urban	154	25.1	25.1
	Rural	459	74.9	100
	Early childhood education	187	30.5	30.5
Field of teacher education	Primary education	201	32.7	63.2
	Secondary education	225	36.3	100
Total		613	100	100

In the present study, school-based practicum mentors were in-service teachers assigned by partner schools to supervise pre-service teachers during their practicum placements. Their responsibilities included classroom observation, lesson-planning guidance, instructional feedback, demonstration teaching, and support for reflective practice. Practicum mentors were generally selected from qualified in-service teachers who held valid teaching qualification certificates and had relevant classroom teaching experience, with selection criteria typically including teaching competence, professional expertise, and the collaborative arrangements between universities and partner schools. Although mentor preparation procedures may vary across institutions, these mentors generally served as the primary school-based supervisors during the practicum period. The present study examined pre-service teachers' perceptions of mentors' naturally occurring leadership behaviors within this supervisory context.

Data were collected between February and June 2025. Ethical approval was obtained from the institutional review boards of the participating universities prior to data collection. All participants took part voluntarily and provided written informed consent. All data were used solely for anonymous academic research purposes, in compliance with relevant ethical and privacy standards.

### 3.2 Measures

All items were rated on a five-point Likert scale ranging from 1 (strongly disagree) to 5 (strongly agree), unless otherwise noted. Higher scores indicate higher levels of the measured construct. Before formal data collection, a pilot study was conducted with a separate subsample to evaluate the psychometric properties of the adapted instruments. Items were revised based on pilot results prior to the main survey.

#### 3.2.1 Transformational leadership

Transformational leadership was measured using the individually focused transformational leadership subscale developed by (Wang and Howell 2010), which is grounded in Bass's (1995) transformational leadership theory and specifically designed to assess leader influence at the individual level. To adapt the instrument for the school-based practicum mentor context, item wording was modified accordingly—for example, references to “the leader” were replaced with “my school-based practicum mentor,” and “work tasks” were replaced with “teaching and academic tasks”—while the original factor structure and response format were retained. The adapted scale comprises 18 items across four dimensions. Pilot results indicated satisfactory reliability and validity (Cronbach's α = 0.856; KMO > 0.80; Bartlett's test of sphericity significant at *p* < 0.001), with a factor structure consistent with the original theoretical dimensions.

#### 3.2.2 Teacher self-efficacy

TSE was assessed using the short form of the Teachers' Sense of Efficacy Scale (TSES-12) developed by (Tschannen-Moran and Hoy 2001). The scale measures TSE across three dimensions: Instructional Strategies (4 items), Classroom Management (4 items), and Student Engagement (4 items), for a total of 12 items. To enhance contextual fit for pre-service teachers on practicum placement, surface-level wording adjustments were made without altering the original factor structure—for example, “your classroom” was changed to “your practicum classroom” and “students” was specified as “students at your practicum school.” Higher total scores indicate higher perceived levels of teaching self-efficacy. Pilot results confirmed acceptable reliability and validity (Cronbach's α = 0.857; KMO > 0.80; Bartlett's test significant at *p* < 0.001), with a clear three-factor structure.

#### 3.2.3 Scholarship of teaching and learning

Scholarship of Teaching and Learning (SoTL) was assessed using the scale developed by (Liu 2021), which was originally designed to evaluate the teaching scholarship of university faculty members. To date, no internationally validated SoTL instrument specifically targeting the practicum context of pre-service teachers has been established; Liu's scale was selected because its dimensional structure closely corresponds to the operationalization of SoTL adopted in this study, and it has undergone preliminary validation in Chinese higher education settings. Contextual adaptations included replacing “university teachers” teaching practice” with “pre-service teachers' practicum teaching practice” to more accurately reflect the participants' task context and stage of professional development. The adapted scale retains the original six dimensions and 23 items. Pilot results demonstrated strong reliability and structural validity (Cronbach's α = 0.922; KMO > 0.80; Bartlett's test significant at *p* < 0.001; all factor loadings > 0.60; cumulative variance explained > 70%). Because the SoTL measure was based on self-report data, the scores should be interpreted as pre-service teachers' perceived SoTL-related competence and practices rather than as direct observational evidence of actual teaching performance. This measurement approach is appropriate for capturing participants' self-perceived engagement in reflective, inquiry-oriented, and improvement-focused teaching practices, but it does not replace external evaluation or classroom observation.

#### 3.2.4 Work engagement

Work engagement was measured using the nine-item Utrecht Work Engagement Scale (Schaufeli et al., 2006), which assesses three dimensions of work-related psychological investment: Vigor (3 items), Dedication (3 items), and Absorption (3 items). In the present study, work engagement specifically refers to pre-service teachers' active psychological investment and sustained participation in practicum teaching and teaching-academic tasks. Pilot results indicated excellent reliability and confirmed the original three-factor structure (Cronbach's α = 0.971; KMO > 0.80; Bartlett's test significant at *p* < 0.001), supporting the suitability of the instrument for use in the main survey.

### 3.3 Data screening and analytical strategy

Prior to the main analyses, the dataset was screened for completeness, response quality, normality, and multicollinearity. All 613 valid questionnaires were complete, with no missing data identified. Univariate normality was examined using skewness and kurtosis values. Skewness values for the four main variables ranged from −0.244 to 0.221, and kurtosis values ranged from −0.745 to 1.131, all within commonly accepted thresholds (|skewness| < 2, |kurtosis| < 7), indicating no serious violation of normality. Multicollinearity was assessed using variance inflation factor (VIF) values. The VIF values for all predictors ranged from 1.093 to 1.403, well below the conventional cutoff value of 10, indicating no meaningful multicollinearity among the study variables.

All analyses were conducted using IBM SPSS Statistics 26.0, the PROCESS macro for SPSS, and IBM SPSS AMOS 26.0, with two-tailed tests at *p* < 0.05.

## 4 Results

This section reports the empirical results based on the formal survey data. Specifically, common method bias, descriptive statistics and correlations, scale reliability, the measurement model, and the moderated mediation model were examined in sequence to test the proposed hypotheses.

### 4.1 Common method bias

Because all data in this study were collected through self-report questionnaires from the same respondents, common method bias (CMB) could not be ruled out. To address this issue, both procedural and statistical remedies were applied. Procedurally, the scales measuring different constructs were arranged separately in the questionnaire, and all responses were collected anonymously in order to reduce the potential influence of social desirability and consistency motives. Statistically, Harman's single-factor test was conducted using an unrotated exploratory factor analysis. The results showed that the first factor accounted for 38.69% of the total variance, which was below the commonly used threshold of 40%, and that 10 factors with eigenvalues greater than 1 were extracted. These findings suggest that common method bias was within an acceptable range and was unlikely to pose a serious threat to the results of this study.

### 4.2 Descriptive statistics and correlation analysis

Table 2 presents the descriptive statistics and correlations among the main variables. The mean scores of the variables ranged from 3.24 to 3.43, and the standard deviations ranged from 0.59 to 1.10. All variables were positively and significantly correlated at the *p* < 0.001 level, providing preliminary support for the hypothesized directions of the relationships. In addition, all correlation coefficients were below commonly accepted critical thresholds, suggesting that there was no serious problem of overlapping associations among the variables.

**Table 2:** Descriptive statistics and correlations among the main variables.

Variables	*M*	*SD*	TL	TSES	SoTL	UWES
TL	3.42	0.60	1			
TSES	3.43	0.59	0.191^**^	1		
SoTL	3.24	1.10	0.537^**^	0.647^**^	1	
UWES	3.32	0.95	0.500^**^	0.286^**^	0.637^**^	1

*p* < 0.001. All tests were two-tailed. ^**^p < 0.01.

### 4.3 Scale reliability

Cronbach's alpha coefficients were calculated to assess the internal consistency of the four scales. As shown in Table 3, all alpha coefficients exceeded 0.80, indicating satisfactory internal reliability for the measures used in this study.

**Table 3:** Reliability of the measurement scales.

Scale name	Number of items	Cronbach's α
TL	18	0.891
TSES	12	0.841
SoTL	23	0.981
UWES	9	0.961

### 4.4 Measurement model

**Figure 2:**
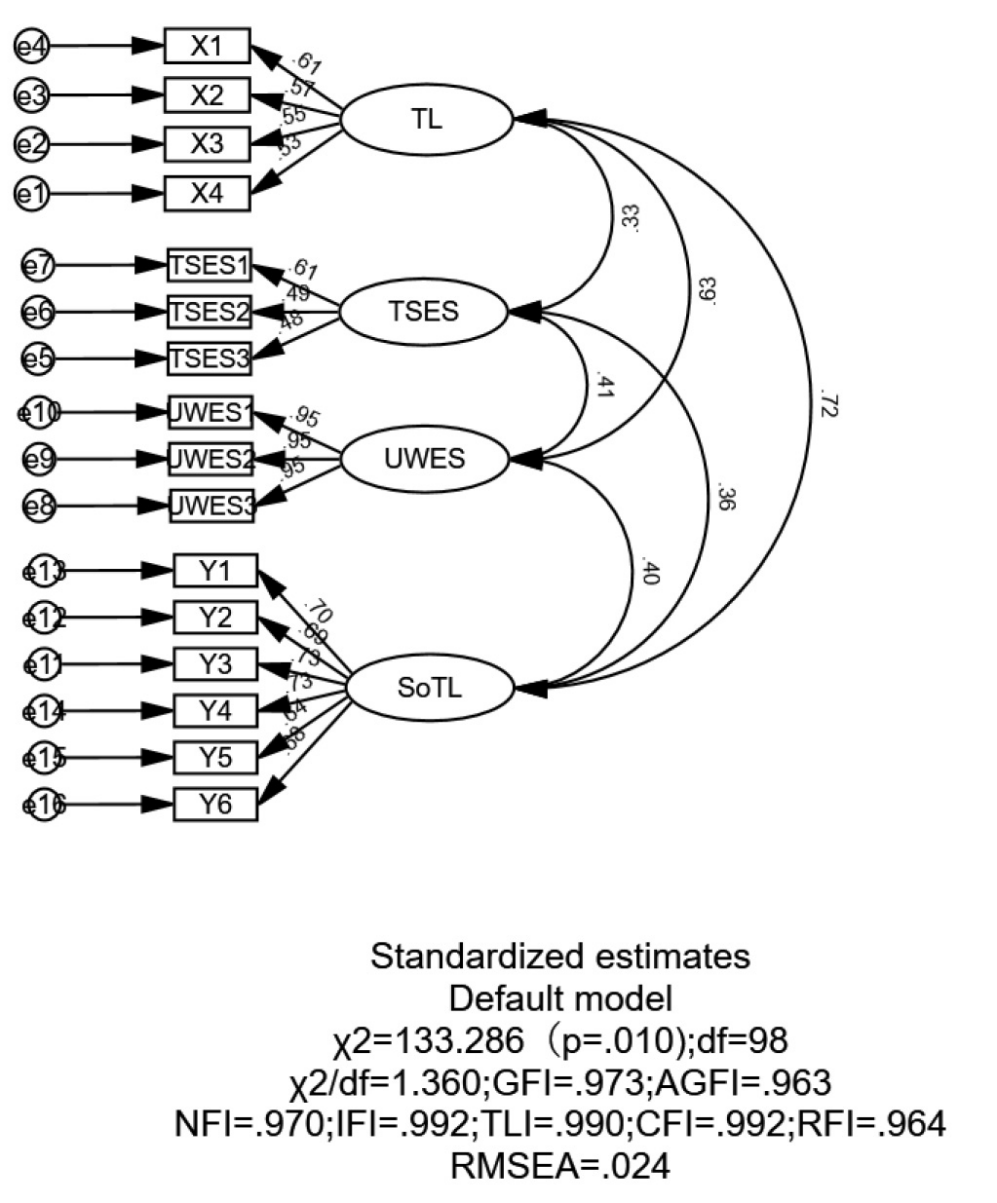
Measurement model.

A confirmatory factor analysis (CFA) was conducted to examine the four-factor measurement model, as shown in Figure 2. The model demonstrated a good fit to the data, with χ^2^/*df* = 1.360, CFI = 0.992, TLI = 0.990, and RMSEA = 0.024. All standardized factor loadings were statistically significant (*p* < 0.001). Two items showed factor loadings of 0.48, which were slightly below the commonly used threshold of 0.50. However, these items were retained because they were theoretically meaningful and their removal did not lead to any substantial improvement in model fit. All remaining items had factor loadings above 0.50, indicating that the overall factor structure was stable. The correlations among the latent variables ranged from 0.33 to 0.72, all of which were below the conventional cutoff value of 0.85, suggesting acceptable discriminant validity among the constructs. Taken together, these results indicate that the measurement model was sufficiently robust for subsequent structural analyses.

### 4.5 Structural model fit

**Figure 3:**
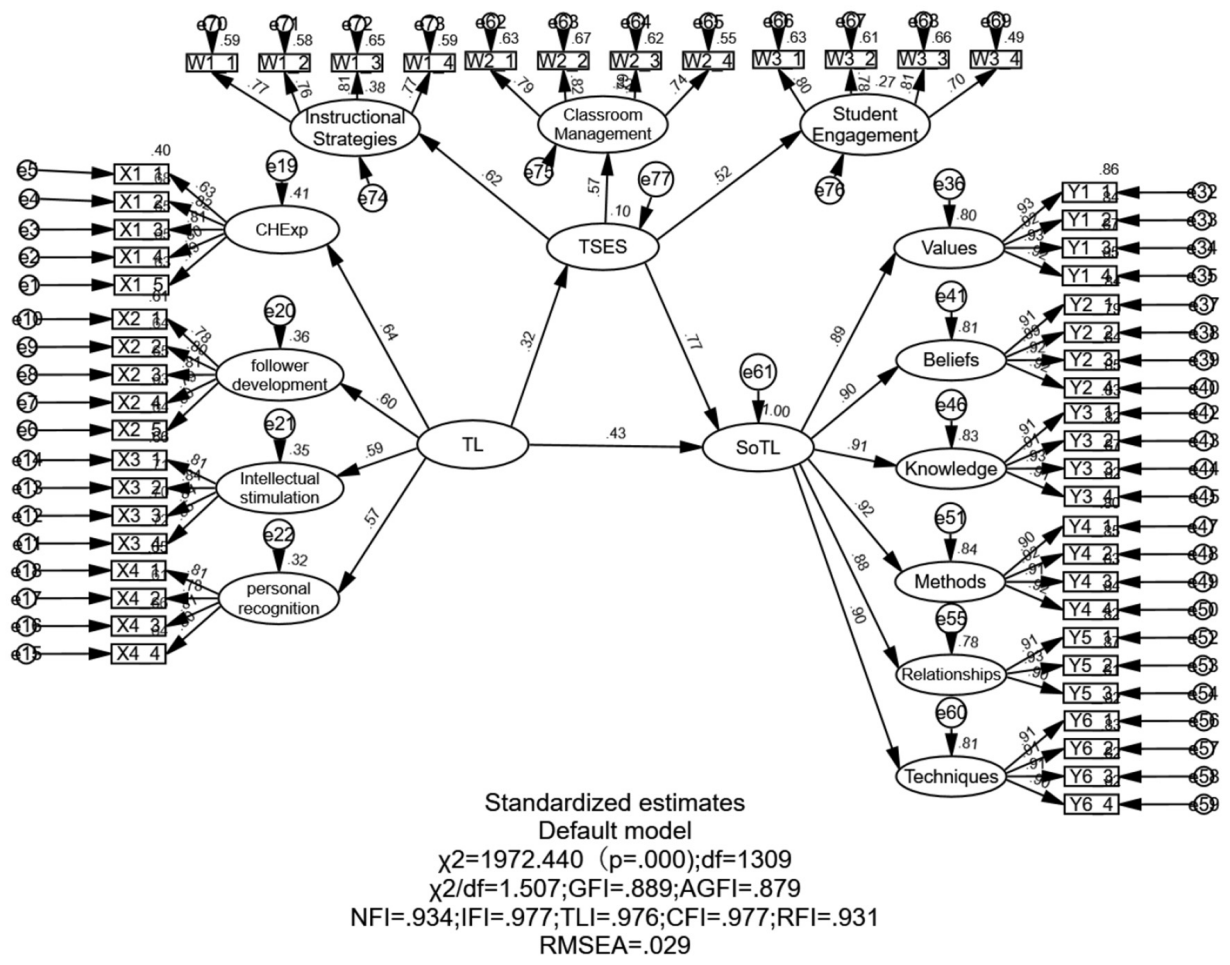
Structural model.

The structural model was then tested using structural equation modeling. As shown in Table 4; Figure 3, the model demonstrated a good fit to the data, with χ^2^/*df* = 1.507, CFI = 0.977, RMSEA = 0.029, and SRMR = 0.0314. These fit indices indicate that the structural model was acceptable and appropriate for further hypothesis testing.

**Table 4:** Fit indices for the structural equation model.

Model	χ^2^	*df*	χ^2^/*df*	*p*	CFI	RMSEA	SRMR
	1972.44	1309	1.507	< 0.001	0.977	0.029	0.0314

### 4.6 Moderated mediation analysis

To examine the proposed moderated mediation model, Hayes' (2018) PROCESS Model 14 was conducted with 5,000 bootstrap resamples. Prior to creating the interaction term, the independent variable, mediator, and moderator were mean-centered in order to reduce multicollinearity and facilitate interpretation.

As shown in Table 5, TL significantly and positively predicted TSES (B = 0.187, SE = 0.039, *p* < 0.001), thereby supporting H2. When predicting SoTL, both TSES (B = 0.890, SE = 0.041, *p* < 0.001) and UWES (B = 0.416, SE = 0.029, *p* < 0.001) had significant positive effects. In addition, the interaction term between TSES and UWES was significant (B = 0.297, SE = 0.043, *p* < 0.001), indicating that UWES significantly moderated the relationship between TSES and SoTL, thus supporting H4. The overall model explained 71.4% of the variance in SoTL (*R*^2^ = 0.714). Furthermore, the direct effect of TL on SoTL remained significant (B = 0.461, 95% CI [0.373, 0.555]), supporting H1.

**Table 5:** Model coefficients for the conditional process analysis.

Antecedents	Consequent					
	M(TSES)			Y(SoTL)		
	Coeff.	SE	*P*	Coeff.	SE	*P*
Constant	−0.641	0.135	< 0.001	1.610	0.155	< 0.001
TL	0.187	0.039	< 0.001	0.461	0.045	< 0.001
TSES				0.890	0.041	< 0.001
UWES				0.416	0.029	< 0.001
Int_1				0.297	0.043	< 0.001
	R-sq = 0.036 *F* = 23.080			R-sq = 0.714 *F* = 380.232		
	*P* < 0 .001			*P* < 0.001		

Unstandardized coefficients (*B*) reported with standard errors (*SE*). Int_1 = TSES × UWES.

**Figure 4:**
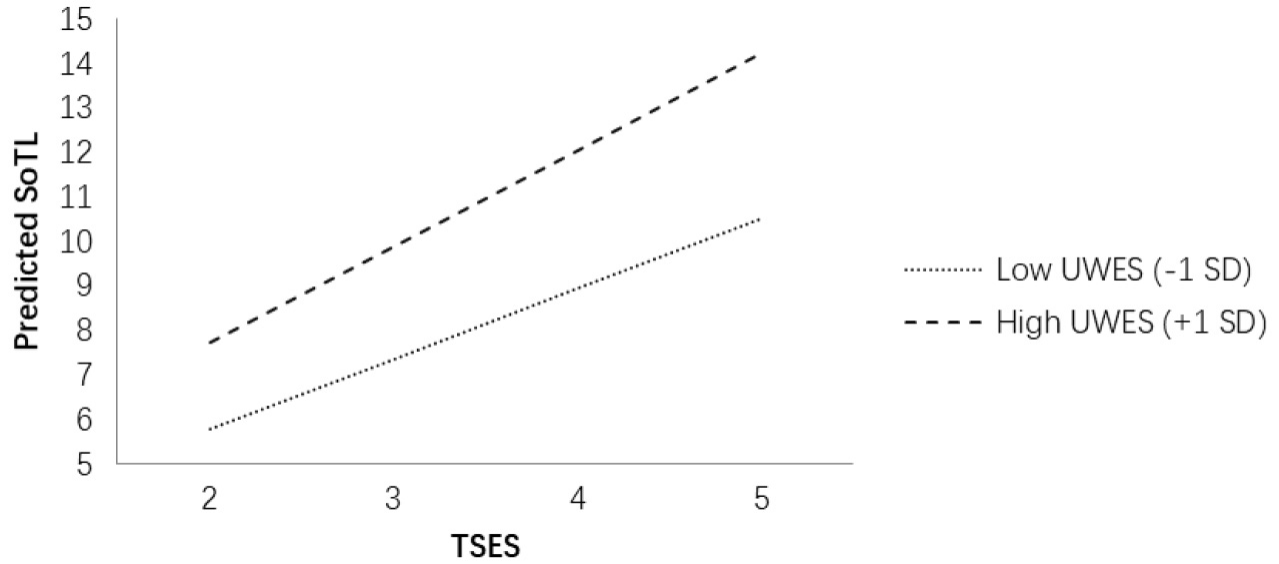
Interaction of teacher self-efficacy and work engagement in predicting SoTL. The x-axis represents TSES, and the y-axis represents predicted SoTL. The steeper slope for the high-UWES group indicates that work engagement strengthens the positive TSES–SoTL relationship.

The simple slope analysis (see Table 6 and Figure 4) further showed that the effect of TSES on SoTL was significant at low (−1 SD), mean, and high (+1 SD) levels of UWES, and that the magnitude of this effect increased as UWES increased, providing additional support for H4. Moreover, the index of moderated mediation was 0.056 (BootSE = 0.014, 95% CI [0.030, 0.083]), and the confidence interval did not include zero. This indicates that the mediating effect of TSES varied significantly across levels of work engagement, confirming the presence of a significant moderated mediation effect. Taken together with the significant direct and interaction effects reported above, these findings support both H3 and H4.

**Table 6:** Simple slopes and conditional indirect effects in the moderated mediation model.

Panel A: Simple slopes of TSES→SoTL at values of UWES				
Moderator value (UWES)	Effect	*SE*	LLCI	ULCI
−0.957	0.607	0.059	0.490	0.723
0	0.890	0.038	0.809	0.972
0.967	1.174	0.058	1.061	1.287
Direct effect of TL on SoTL	*SE*	*p*	LLCI	ULCI
0.461	0.045	< .001	0.373	0.550
Panel B. Conditional indirect effects of TL on SoTL via TSES at values of UWES				
**Indirect effect(X → M → Y)**	**Effect**	***SE***	**LLCI**	**ULCI**
−0.957	0.114	0.027	0.063	0.17
0	0.167	0.034	0.097	0.234
0.967	0.22	0.045	0.127	0.305
Index of moderated mediation	Effect	*SE*	LLCI	ULCI
	0.056	0.014	0.03	0.083

Conditional effects are evaluated at UWES = −1 *SD*, Mean, and +1 *SD*. Bootstrap CIs based on 5,000 resamples.

## 5 Discussion

### 5.1 Discussion of findings

The present study constructed and tested a moderated mediation model examining the pathway from school-based practicum mentors' transformational leadership to pre-service teachers' SoTL, with TSE as the mediator and work engagement as the moderator. All four hypotheses were supported. Beyond confirming the direct facilitative effect of transformational leadership on SoTL, the findings reveal the critical mediating role of TSE in this process and the boundary condition function of work engagement in translating efficacy beliefs into sustained professional behavior. Taken together, these results indicate that school-based practicum mentors are not merely supervisors of practical skills; they are also an important contextual source shaping pre-service teachers' TSE formation and professional competence development. Nevertheless, the uniformly positive pattern of findings should be interpreted with caution. Given the cross-sectional and self-report nature of the data, the results are better understood as theoretically guided associations rather than definitive evidence of causal effects. It is also possible that pre-service teachers who reported higher levels of SoTL tended to evaluate their mentor relationships, efficacy beliefs, and practicum engagement more positively. Therefore, while the findings are consistent with and supportive of the proposed theoretical model, future research employing longitudinal or experimental designs is needed to establish more robust causal conclusions.

Several boundary conditions may also qualify the present findings. The positive effect of transformational leadership on TSE may be attenuated in contexts characterized by significant power imbalances or institutional constraints such as heavy mentor workloads or rigid evaluation systems. The mediating role of TSE may be less pronounced among pre-service teachers with prior teaching experience or stronger dispositional resilience. Furthermore, in highly structured or examination-oriented school settings, even highly engaged pre-service teachers may face institutional barriers that constrain the translation of efficacy beliefs into SoTL practices. These considerations suggest that the relationships identified in the present study should be understood as context-sensitive rather than universally generalizable.

First, the results support H1, demonstrating that school-based practicum mentors' transformational leadership is significantly and positively associated with pre-service teachers' SoTL. This finding indicates that mentor behaviors enacted during the practicum—including offering an inspiring vision, providing individualized care, and stimulating intellectual inquiry—not only shape pre-service teachers' professional attitudes but also directly promote the development of their capacity for pedagogical inquiry and reflective practice. This result is interpretable within the framework of social cognitive theory: (Bandura 1997) argued that individual behavior is shaped not only by internal cognitive processes but also by direct environmental influences. In the practicum context, mentors who model teaching innovation, provide constructive feedback, and cultivate a high-expectation atmosphere directly influence pre-service teachers' behavioral orientations toward teaching. This finding extends prior research, which has primarily linked transformational leadership to emotional commitment and job satisfaction (Fisher, 2023; Ren and Li, 2024), by demonstrating that mentor leadership behaviors in teacher education contexts can exert equally meaningful effects on SoTL—a more professionally specialized outcome variable. Importantly, this finding carries direct implications for the evidence-based design of practicum supervision systems: systematically embedding transformational leadership behaviors into mentor training programs may constitute an effective, empirically grounded pathway for cultivating pre-service teachers' SoTL.

Second, the results support H2, indicating that school-based practicum mentors‘ transformational leadership significantly enhances pre-service teachers' TSE. This finding further validates the “environment–belief” pathway central to social cognitive theory. (Bandura 1997) identified mastery experiences, vicarious experiences, verbal persuasion, and physiological states as the four principal sources of self-efficacy. Mentors' feedback and role modeling constitute important instances of these sources. More specifically, mentors' inspirational motivation and intellectual stimulation behaviors are likely to function as vicarious experience and verbal persuasion, respectively—two of Bandura's core efficacy sources—thereby illuminating the specific psychological pathway through which transformational leadership enhances TSE in the practicum context. The positive encouragement, pedagogical modeling, and problem-oriented guidance provided by mentors during the practicum strengthen pre-service teachers' confidence in their own teaching capabilities. This is consistent with (Holzberger et al. 2013) findings on the relationship between teacher support and self-efficacy, and aligns with the growing body of research emphasizing the role of leadership support in shaping teachers' efficacy beliefs (Xie et al., 2022). Notably, the present study was conducted in an informal leadership context—one in which mentors hold no formal administrative authority—yet mentor behaviors still exerted a significant effect on TSE. This suggests that leadership influence is not confined to formal hierarchical structures but can operate through the quality of interpersonal interaction.

Third, the results support H3, demonstrating that TSE significantly mediates the relationship between transformational leadership and SoTL. The persistence of a significant direct effect indicates that this is a case of partial mediation. This finding delineates a clear “leadership–belief–competence” psychological mechanism: according to social cognitive theory, the initiation and maintenance of behavior depends on individuals' judgments about their own capabilities. Even when external support is available, the absence of efficacy beliefs may prevent that support from translating into sustained behavioral engagement; conversely, when TSE is strengthened, individuals are more likely to embrace challenging tasks and persevere in the face of difficulty. This is consistent with Klassen and Tze's (2014) findings linking self-efficacy to teacher performance, and suggests that TSE functions as a key psychological conduit through which mentor leadership shapes pre-service teachers' professional practice. Crucially, the partial mediation pattern suggests that mentors' transformational leadership influences SoTL not only through TSE but also through additional pathways—such as emotional motivation or normative role modeling—that operate independently of efficacy beliefs. This opens productive directions for future research aimed at constructing more comprehensive multiple-mediation models.

Fourth, the results support H4, showing that work engagement positively moderates the relationship between TSE and SoTL, such that the positive effect of TSE on SoTL is stronger when work engagement is higher. This finding reveals an important boundary condition. While social cognitive theory emphasizes the belief–behavior link, it does not fully specify the conditions under which efficacy beliefs are most likely to be converted into action. The present study demonstrates that work engagement—as an expression of individuals‘ psychological energy and sustained attentional investment—constitutes a critical amplifying factor in this conversion process. Pre-service teachers who are highly engaged in their practicum tasks are more inclined to translate their capability beliefs into active pedagogical reflection and innovative experimentation; conversely, even when TSE is high, behavioral translation may be attenuated under conditions of low engagement. This finding aligns with Schaufeli et al.'s (2006) conceptualization of work engagement as a driver of performance, and responds to the growing emphasis in the teacher education literature on the importance of positive psychological resources. In sum, the present study not only confirms the role of the efficacy mechanism but further specifies that the behavioral effects of efficacy beliefs are contingent upon individuals' level of psychological investment.

### 5.2 Theoretical contributions

The present study makes three principal theoretical contributions.

First, the study extends the boundary conditions of transformational leadership theory. Existing research has predominantly focused on formal leaders—such as school principals and institutional administrators—who hold positional authority, with comparatively little attention directed toward non-positional mentors in practicum contexts. The present study demonstrates that transformational leadership behaviors enacted by school-based practicum mentors—individuals without formal administrative authority—can exert equally meaningful effects on pre-service teachers' professional competence. This finding expands the situational applicability of transformational leadership theory and opens new avenues for examining informal, non-positional leadership in teacher education settings.

Second, the study identifies an important developmental source of TSE during initial teacher education. Although TSE has been widely recognized as a critical psychological resource for teacher professional development, prior research has tended to focus on its downstream outcomes or to examine it within general school organizational contexts. In contrast, the present study situates TSE within the practicum—a pivotal professional experience—and demonstrates that school-based practicum mentors‘ transformational leadership behaviors significantly shape pre-service teachers' judgments about their own teaching capabilities. In doing so, the study provides new empirical evidence on how TSE is formed during the initial phase of teacher education. Put differently, the study moves beyond asking what TSE does, to addressing how and through what kinds of supportive relationships TSE comes to be formed in the first place.

Third, the study introduces work engagement as a key boundary condition variable, revealing the contextual amplification mechanism through which TSE is converted into SoTL. The findings demonstrate that TSE does not automatically translate into sustained professional practice; rather, its behavioral effects are more pronounced when individuals' levels of work engagement are high. This finding enriches the dynamic interpretation of the belief–behavior relationship within social cognitive theory and advances understanding of the conditions under which TSE is most effectively converted into professional growth in teacher education contexts.

### 5.3 Practical implications

The findings of the present study carry several implications for teacher education reform and the professional development of school-based practicum mentors.

First, teacher education institutions should view the practicum not merely as a placement requirement, but as a critical context for cultivating pre-service teachers' teacher self-efficacy and professional growth. The present findings suggest that school-based practicum mentors do more than provide practical instructional guidance; through transformational leadership behaviors, they also shape pre-service teachers' efficacy beliefs and, in turn, their development of SoTL. Mentor training programs should therefore explicitly foster mentors' capacity to provide inspirational feedback, individualized guidance, reflective support, and opportunities for pedagogical experimentation, so that the practicum can function not only as a site of practice, but also as a developmental context for strengthening TSE. Systematically embedding these leadership-oriented mentoring behaviors into mentor preparation programs may contribute to more theoretically grounded and practically effective practicum supervision models.

Second, teacher education programs should attend explicitly to the development of pre-service teachers' TSE during the practicum. Practicum placements should be designed to provide pre-service teachers with abundant opportunities for mastery experience, vicarious observation, supportive feedback, and positive interpersonal interaction—all of which strengthen their confidence in and sense of control over teaching tasks. Teacher education programs need to attend not only to what tasks pre-service teachers complete, but also to how they come to understand their own capabilities and build confidence in teaching. Only when pre-service teachers have developed robust TSE are external forms of support likely to be effectively converted into sustained professional behavior and competence growth.

Third, teacher education programs should create practicum environments that are conducive to high levels of work engagement. The present findings indicate that work engagement amplifies the conversion of TSE into SoTL. Providing pre-service teachers with clear professional goals, meaningful growth opportunities, and a strong sense of active participation in practice are likely to enhance their psychological investment in the practicum, and thereby maximize the professional development returns of their efficacy beliefs.

### 5.4 Limitations and future directions

Despite its contributions, the present study has several limitations that should be acknowledged.

First, the reliance on cross-sectional data limits the rigor of causal inference among the study variables. Specifically, reversed causal directions cannot be ruled out; for example, pre-service teachers with higher levels of SoTL may be more likely to perceive mentor behaviors positively, rather than mentor leadership unidirectionally shaping SoTL. Future research would benefit from adopting longitudinal tracking or experimental designs to strengthen causal interpretations. In particular, intervention studies focusing on mentor training would be valuable for examining whether systematically cultivating transformational leadership behaviors among practicum mentors can produce sustained improvements in pre-service teachers' TSE and SoTL. Such research would move the field from descriptive mechanism analysis toward stronger causal verification and evidence-based practicum reform.

Second, the study relied on single-source self-report data, which may inflate associations among the study variables and limit the assessment of actual SoTL-related practice. In particular, the measurement of SoTL may reflect pre-service teachers' perceived engagement in reflective and inquiry-oriented practices rather than independently observed teaching behavior. Future studies should incorporate multiple data sources, such as mentor evaluations, practicum records, student feedback, classroom observations, teaching portfolios, or peer evaluations, to provide a more robust assessment of SoTL development.

Third, the present study focused on individual-level psychological mechanisms and did not fully examine broader contextual factors that may shape practicum experiences. For example, school climate, mentor–mentee matching procedures, institutional practicum policies, and the quality of university–school collaboration may influence how mentor leadership is perceived and how efficacy beliefs are translated into SoTL. Future research could adopt multilevel designs to examine how individual-level psychological processes interact with school- or program-level contextual conditions.

Fourth, the sample was drawn from universities in western China, which may limit the generalizability of the findings. Future research should extend to diverse cultural and institutional settings, and incorporate additional variables—such as professional identity or learning goal orientation—to construct more comprehensive models of SoTL development.

## 6 Conclusion

The present study constructed and empirically validated a moderated mediation model that systematically elucidates the mechanisms through which school-based practicum mentors' transformational leadership influences pre-service teachers' SoTL. The findings demonstrate that transformational leadership not only exerts a direct positive effect on SoTL but also produces a significant indirect effect by enhancing pre-service teachers' TSE. Work engagement, in turn, functions as a critical boundary condition that amplifies the conversion of TSE into SoTL.

These findings indicate that the development of pre-service teachers' SoTL is not solely dependent on external practical support, but is jointly shaped by their internal efficacy beliefs and psychological investment in professional tasks. Grounded in social cognitive theory and transformational leadership theory, the study delineates a dynamic developmental pathway—“contextual support → efficacy beliefs → behavioral translation”—through which professional competence develops in pre-service teachers.

Of particular significance, the present study situates the formation of TSE within the practicum context, addressing a notable gap in the existing literature regarding the sources and developmental pathways of TSE during initial teacher education. The findings offer new empirical evidence on how TSE is formed through mentoring relationships during the practicum stage—moving the field's understanding beyond what TSE does, toward a clearer account of how and where it develops.

Against the backdrop of teacher education reform and the broader imperative to develop high-quality teachers, the present study integrates transformational leadership theory with social cognitive theory to provide a more complete and dynamic explanatory framework for understanding the psychological processes underlying pre-service teacher professional competence development. In doing so, it establishes an empirical foundation for future intervention research and evidence-based practicum system design.

## Data Availability

The raw data supporting the conclusions of this article will be made available by the authors, without undue reservation.

## References

[B1] Bandura A. (1997). Self-Efficacy: The Exercise of Control. New York, NY: W.H. Freeman.

[B2] Bass B. M. (1985). Leadership and Performance Beyond Expectations. New York, NY: Free Press.

[B3] Bass B. M. (1995). Theory of transformational leadership redux. Leadersh. Q. 6, 463–478. doi: 10.1016/1048-9843(95)90021-7

[B4] Bass B. M. Riggio R. E. (2006). Transformational Leadership, 2nd edn. New York, NY: L. Erlbaum Associates. doi: 10.4324/9781410617095

[B5] Boyer E. L. (1990). Scholarship Reconsidered: Priorities of the Professoriate. Princeton, NJ: Princeton University Press.

[B6] Cai Y. Tang R. (2021). School support for teacher innovation: mediating effects of teacher self-efficacy and moderating effects of trust. Thinking Skills Creativity 41:100854. doi: 10.1016/j.tsc.2021.100854

[B7] Cai Y. Wang L. Bi Y. Tang R. (2022). How can the professional community influence teachers' work engagement? The mediating role of teacher self-efficacy. Sustainability 14:10029. doi: 10.3390/su141610029

[B8] Fisher K. (2023). Reimagining mentorship for doctoral student success: the potential utility of transformational leadership practices. Int. J. Leadersh. Educ. 1–17. doi: 10.1080/13603124.2023.2295457

[B9] Galamay-Cachola S. Aduca C. M. Calauagan F. (2018). Mentoring experiences, issues, and concerns in the student-teaching program: towards a proposed mentoring program in teacher education. IAFOR J. Educ. 6, 7–24. doi: 10.22492/ije.6.3.01

[B10] Goldshaft B. Sjølie E. (2024). Creating communicative learning spaces in initial teacher education (ITE) with observation-grounded co-mentoring practices. Prof. Dev. Educ. 50, 533–550. doi: 10.1080/19415257.2024.2337772

[B11] Gu J. He C. Liu H. (2015). Supervisory styles and graduate student creativity: the mediating roles of creative self-efficacy and intrinsic motivation. Stud. High. Educ. 42, 721–742. doi: 10.1080/03075079.2015.1072149

[B12] Han J. Liu N. Wang F. (2022). Graduate students' perceived supervisor support and innovative behavior in research: the mediation effect of creative self-efficacy. Front. Psychol. 13:875266. doi: 10.3389/fpsyg.2022.875266 35783747 PMC9249313

[B13] Hassan R. S. Amin H. M. G. Ghoneim H. (2024). Decent work and innovative work behavior of academic staff in higher education institutions: the mediating role of work engagement and job self-efficacy. Humanit. Soc. Sci. Commun. 11, 702–720. doi: 10.1057/s41599-024-03177-0

[B14] Hayes A. F. (2018). Introduction to Mediation, Moderation, and Conditional Process Analysis: A Regression-Based Approach, 2nd edn. New York, NY: Guilford Press.

[B15] Hidayat R. Patras Y. E. (2024). Teacher innovativeness: the effect of self-efficacy, transformational leadership, and school climate. J. Pedagogical Res. 8, 208–222. doi: 10.33902/JPR.202424547

[B16] Holzberger D. Philipp A. Kunter M. (2013). How teachers' self-efficacy is related to instructional quality: a longitudinal analysis. J. Educ. Psychol. 105, 774–786. doi: 10.1037/a0032198

[B17] Johnson J. L. (2022). Teacher self-efficacy and teacher work engagement for expats at international K12 schools in China: a correlation analysis. Int. J. Educ. Res. Open 3:100176. doi: 10.1016/j.ijedro.2022.100176

[B18] Klassen R. M. Tze V. M. C. (2014). Teachers' self-efficacy, personality, and teaching effectiveness: a meta-analysis. Educ. Res. Rev. 12, 59–76. doi: 10.1016/j.edurev.2014.06.001

[B19] Kuhn C. Hagenauer G. Gröschner A. Bach A. (2024). Mentor teachers' motivations and implications for mentoring style and enthusiasm. Teach. Teach. Educ. 139:104441. doi: 10.1016/j.tate.2023.104441

[B20] Kuswandono P. (2017). Mentor teachers‘ voices on pre-service English teachers' professional learning. Indones. J. Appl. Ling. 6, 213–221. doi: 10.17509/ijal.v6i2.4846

[B21] Lefteri A. Menon M. E. (2025). Transformational and transactional school leadership as predictors of teacher self-efficacy. Stud. Educ. Eval. 86:101476. doi: 10.1016/j.stueduc.2025.101476

[B22] Leithwood K. Jantzi D. (2006). Transformational school leadership for large-scale reform: effects on students, teachers, and their classroom practices. Sch. Eff. Sch. Improv. 17, 201–227. doi: 10.1080/09243450600565829

[B23] Liu G. (2021). Research on the Core Capability of University Teachers' Scholarship of Teaching and Learning and Strategy for Improvement (doctoral dissertation). China University of Mining and Technology, CNKI, Beijing, China.

[B24] Ma K. McMaugh A. Cavanagh M. (2022). Changes in pre-service teacher self-efficacy for teaching in relation to professional experience placements. Aust. J. Educ. 66, 57–72. doi: 10.1177/00049441211060474

[B25] Orland-Barak L. Wang J. (2021). Teacher mentoring in service of preservice teachers' learning to teach: conceptual bases, characteristics, and challenges for teacher education reform. J. Teach. Educ. 72, 86–99. doi: 10.1177/0022487119894230

[B26] Ren J. Li X. (2024). Mentor support and postgraduate research ability: the role of research self-efficacy and academic atmosphere. Asia Pac. J. Educ. 1–15. doi: 10.1080/02188791.2024.2364648

[B27] Scandura T. A. Williams E. A. (2004). Mentoring and transformational leadership: the role of supervisory career mentoring. J. Vocational Behav. 65, 448–468. doi: 10.1016/j.jvb.2003.10.003

[B28] Schaufeli W. B. Bakker A. B. Salanova M. (2006). The measurement of work engagement with a short questionnaire: a cross-national study. Educ. Psychol. Meas. 66, 701–716. doi: 10.1177/0013164405282471

[B29] Tschannen-Moran M. Hoy A. W. (2001). Teacher efficacy: capturing an elusive construct. Teach. Teach. Educ. 17, 783–805. doi: 10.1016/S0742-051X(01)00036-1

[B30] Uppathampracha R. Liu G. (2022). Leading for innovation: self-efficacy and work engagement as sequential mediation relating ethical leadership and innovative work behavior. Behav. Sci. 12:266. doi: 10.3390/bs12080266 36004837 PMC9405150

[B31] Wang X.-H. F. Howell J. M. (2010). Exploring the dual-level effects of transformational leadership on followers. J. Appl. Psychol. 95, 1134–1144. doi: 10.1037/a0020754 20718529

[B32] Xie Z. Wu R. Liu H. Liu J. (2022). How does teacher-perceived principal leadership affect teacher self-efficacy between different teaching experiences through collaboration in China? A multilevel structural equation model analysis based on threshold. Front. Psychol. 13:933838. doi: 10.3389/fpsyg.2022.933838 36092083 PMC9453751

[B33] Yilmaz B. Tore E. (2025). The mediating role of self-efficacy in the effect of school principals‘ transformational leadership characteristics on teachers' innovative work behavior. Leadersh. Policy Sch. 1–20. doi: 10.1080/15700763.2025.2462741

[B34] Yong P. Peng Y. (2025). Strategies for enhancing the scholarship of teaching and learning among primary and secondary school teachers: a grounded theory analysis. Front. Educ. 10:1542528. doi: 10.3389/feduc.2025.1542528

[B35] Zee M. Koomen H. M. Y. (2016). Teacher self-efficacy and its effects on classroom processes, student academic adjustment, and teacher well-being: a synthesis of 40 years of research. Rev. Educ. Res. 86, 981–1015. doi: 10.3102/0034654315626801

